# AGER promotes proliferation and migration in cervical cancer

**DOI:** 10.1042/BSR20171329

**Published:** 2018-01-30

**Authors:** Xuejie Zhu, Lulu Zhou, Ruyi Li, Qi Shen, Huihui Cheng, Zongji Shen, Haiyan Zhu

**Affiliations:** 1 Department of Obstetrics and Gynecology, The First Affiliated Hospital of Soochow University, Suzhou, China; 2 Department of Obstetrics and Gynecology, The Second Affiliated Hospital of Wenzhou Medical University, Wenzhou, China

**Keywords:** AGER, cervical cancer, migration, proliferation

## Abstract

The receptor for advanced glycation end products (AGER) is an oncogenic transmembranous receptor up-regulated in various human cancers. We have previously reported that AGER was overexpressed in squamous cervical cancer. However, mechanisms of AGER involved in the progression of cervical cancer are unknown. In the present study, we investigated the effects of AGER on biological behavior, including proliferation, apoptosis, and migration using multiple biological approaches. AGER protein primarily localized in the cytoplasm and cytomembrane of cervical squamous cancer cells. Blockage of AGER with multiple siRNAs suppressed proliferation, stimulated apoptosis, inhibited migration of cervical squamous cancer cells. Conversely, overexpression of AGER increased cell proliferation, migration, and inhibited cell apoptosis. These results indicate that AGER promotes proliferation, migration, and inhibits apoptosis of squamous cervical cancer and might function as a tumor promoter in cervical cancer. Our study provides novel evidence for a potential role of AGER in bridging human papillomavirus (HPV)-induced inflammation and cervical cancer.

## Introduction

Cervical cancer remains the fourth most common cause of cancer-related deaths in women worldwide, with an estimated 527600 new cases and 265700 deaths in 2012 [1]. High risk human papillomavirus (HPV) is considered as a principal etiologic agent for cervical cancer, however, only a small fraction of women exposed to this virus develop cancer, implying that other factors contribute to cervical carcinogenesis [2,3]. It is now well established that inflammation is a critical component of tumor development and progression [4,5]. As well as its direct oncogenic effects, ongoing inflammation induced by persistent HPV infections also is a driving force that accelerates cervical cancer formation [6,7]. In the context of cancer development, inflammation stimulated by virus infection is highly involved in the control of various physiological functions, including increasing cell proliferation and survival, promoting angiogenesis and metastasis, subverting adaptive immune responses, and altering responses to hormones and chemotherapeutic agents [7].

The receptor for advanced glycation end products (AGER), a member of the immunoglobulin superfamily of cell surface molecules, has been well known as a promoter of inflammation [8]. AGER is low or negatively expressed in normal tissues, but increases quickly at sites of inflammation, largely on inflammatory and epithelial cells [9,10]. It drives the strength and maintenance of an inflammatory reaction during tumor promotion and bridges chronic inflammation and cancer [11]. AGER engagement activates multiple intracellular signaling mechanisms that fuel chronic inflammatory conditions leading to malignant transformation [9,10]. Indeed, AGER has been widely reported being highly expressed in various types of cancer, including ovarian cancer [12], breast cancer [13], gastric cancer [14], and endometrial cancer [15]. It is well documented that AGER plays specific roles in the modulation of several cellular events, including proliferation [16], cell motility [17], and angiogenesis [18,19]. Our previous study reported that AGER was progressively up-regulated from cervicitis to cervical intraepithelial neoplasia and cancer [20]. Furthermore, high AGER protein levels in squamous cervical cancer significantly correlated with tumor differentiation [20]. However, its precise mechanism involved in the carcinogenesis of cervix remains unclear. In the present study, we investigated the effects of AGER on the regulation of biological behavior, including cell proliferation, apoptosis, and migration in squamous cervical cancer. The aim of the present study was to establish the possible role of AGER during cervical cancer development and progression.

## Materials and methods

### Cell lines and cell culture

Human cervical cancer cell lines, SiHa, Caski, C33A, and MS751, were purchased from Shanghai Cell Biology Medical Research Institute, Chinese Academy of Sciences. SiHa, C33A, and MS751 cells were maintained in Dulbecco’s modified Eagle’s medium (DMEM) (Invitrogen, NY, U.S.A.) containing 10% FBS, penicillin, and streptomycin at 37°C in a 5% CO_2_ incubator. Caski was cultured in RPMI-1640 (Invitrogen, NY, U.S.A.) containing 10% FBS, penicillin, and streptomycin in 5% CO_2_ at 37°C.

### Immunocytochemical analysis

Immunocytochemical staining for AGER detection was evaluated with routine procedures using anti-AGER rabbit antibody (Santa Cruz, U.S.A.), at 4°C overnight and biotinylated goat anti-rabbit antibody for 30 min at 37°C. The cells were counterstained with Mayer Hematoxylin to enhance nuclear detection, dehydrated, and mounted in distrene dibutylphthalate xylene. Immunostaining of the negative control was incubated with PBS in the absence of primary antibody.

### RNA extraction and quantitative real-time PCR

The mRNA levels were measured by quantitative real-time PCR (qRT-PCR). Total RNA was isolated from cervical cancer cells using TRIzol reagent according to the manufacturer’s instructions (Invitrogen, NY, U.S.A.). Total RNA (2 μg) was reverse transcribed into cDNA using random primers and M-MLV reverse transcriptase from Invitrogen Life Technology (NY, U.S.A.). qRT-PCR was performed using Power SYBR Green PCR Mix from Life Technologies. Primer pair specificity was determined by generation of a single peak for dissociation curve at the end of real-time PCR cycling program. All experiments were performed in triplicates. GAPDH was used as the internal control. The primers used in the study are as follows: AGER, forward primer, 5′-TCATTGGGGTCATCTTGT-3′; reverse primer: 5′-TACTACTCTCGCCTGCCT-3′; GAPDH, forward primer, 5′-AAGAAGGTGGTGAAGCAGG-3′; reverse primer: 5′-GTCAAAGGTGGAGGAGTGG-3′.

### Western blot analysis

Samples were homogenized and lysed in Laemmli buffer with a cocktail of protease inhibitors. The total protein concentrations were quantitated by the BCA protein assay (Thermo Scientific, IL, U.S.A.). Equal amounts of total protein were resolved by SDS/PAGE, transferred on to a nitrocellulose membrane under constant voltage and blocked with TBS with tween (TBST) containing 5% non-fat dried milk. Primary antibodies (AGER, 1:1000, Santa Cruz, CA, U.S.A.; GAPDH, 1:2000, Santa Cruz, CA, U.S.A.; GFP, 1:1000, Cell Signaling, MA, U.S.A.) and secondary antibodies were diluted in TBST and applied with a washing step in between. Proteins were detected using the Amersham ECL Western blotting detection kit (GE Healthcare, NJ, U.S.A.).

### Construction of lentiviruses

GFP-AGER cDNA was subcloned into pLenti-C-mGFP vector (Origene, MD, U.S.A.) *in vitro*. After confirmation using gene sequencing, the pLenti-C-mGFP-AGER plasmid (LV-AGER) and matched pLenti-C-mGFP vector (LV-vector) were co-transfected together with two packaging vectors psPAX2 and pMD2.G into 293T cells. Lentiviral particles were harvested and filtered to infect cervical cancer cell lines.

### Knockdown analysis using AGER siRNAs

Cells were seeded at 30–50% confluence in six-well plates. AGER was transiently silenced by using two different siRNAs targetting AGER (AGER-siRNA-1, -2) (GenePharma, Shanghai, China). A silencer negative transcription control (siRNA-NC) (GenePharma, Shanghai, China) was used in each experiment. Transfection was performed using Lipofectamine RNAiMAX (Invitrogen, NY, U.S.A.) according to the manufacturer’s instructions. Forty-eight hours after transfection, whole-cell lysates were prepared for further analysis by Western blot and *in vitro* CCK-8 assay as well as transwell migration assay as described below.

### CCK-8 assay

CCK-8 assay was performed to determine the effect of AGER expression on cell proliferation in cervical cancer cells. Cells were seeded in 96-well plates (5 × 10^4^ cells/well). After transfection, CCK-8 solution (10 μl/well) was added and incubated at 37°C for 2 h in a humidified incubator. The absorbance value was measured at 450 nm wavelength on a Biotek plate reader (Bio–Rad, U.S.A.). The experiments were repeated three times.

### Flow cytometry

Cells were plated at 5 × 10^5^ cells/dishes into 60-mm dishes. After reaching 70–80% confluence during exponential growth, cells were harvested, washed with cold PBS, and resuspended with binding buffer at a concentration of 1 × 10^6^ cell/ml. Then the cells were double-stained with annexin V-FITC/propidium iodide or PE/7-AAD according to the manufacturer’s protocol (BD Pharmingen, CA, U.S.A.). The percentage of apoptotic cells were detected by flow cytometry after staining. The experiment was repeated three times.

### Transwell migration assay

Cell migration assays were performed in 24-well transwells with 8-μm pore polycarbonate membranes (BD Biosciences, San Diego, CA). Cells at a density of 15000 cells/well in serum-free medium were seeded in the upper insert in triplicates after transfection. The lower chamber was filled with medium containing 10% FBS as a chemoattractant. After incubation in 5% CO_2_ at 37°C for 24 h, the cells that did not penetrate the polycarbonate membrane at the bottom of the chamber were removed with a cotton swab. Then the cells that had invaded through the membrane to the lower surface were fixed with methanol for 20 min and stained with 1% Crystal Violet for 10 min. Five vision fields were selected randomly under a microscope (Nikon, Japan) with 100× magnification, and the number of cells that penetrated the membrane was counted.

### Statistical analysis

Two-tailed Student’s *t*test was used to compare the means of two groups. One-way ANOVA with Tukey–Kramer post hoc test was used for analyzing data when means from more than two groups were compared. *P*<0.05 was considered to be statistically significant. All the statistical analysis was performed with SPSS 17.0 statistical software.

## Results

### The localization of AGER protein in cervical squamous cancer cells

**Figure 1:**
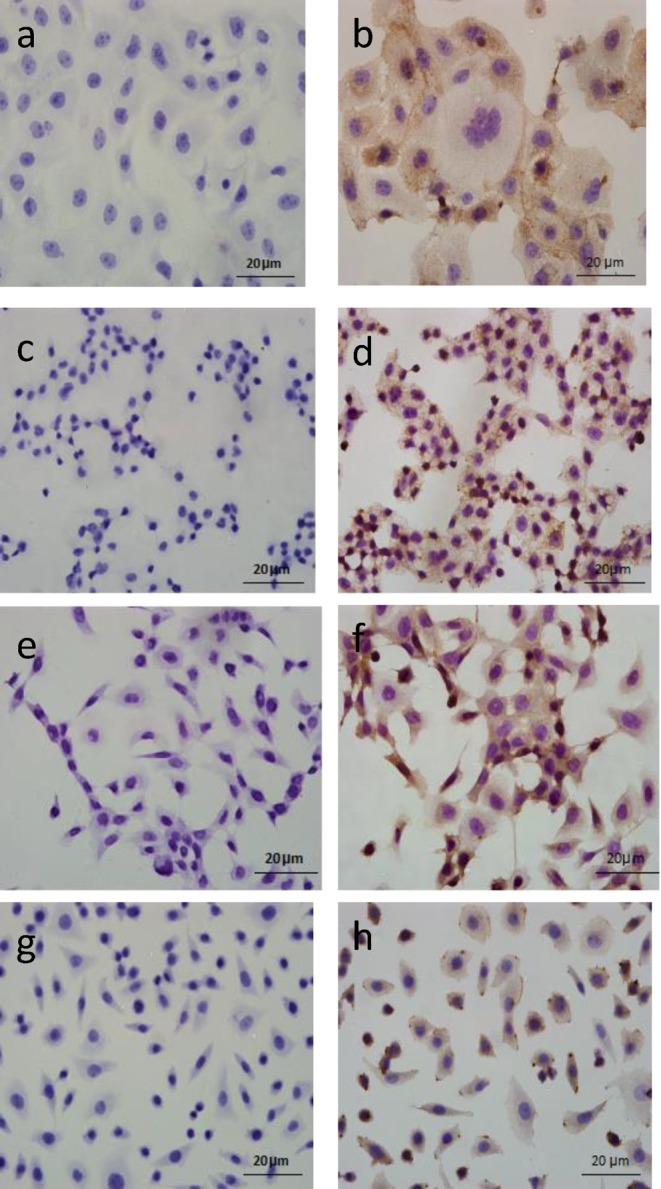
Immunocytochemical staining of AGER in cervical squamous cancer cells (**a**) Negative control in MS751 cells (SP staining, ×400). (**b**) Positive staining of AGER protein in MS751 cells (SP staining, ×400). (**c**) Negative control in C33A cells (SP staining, ×400). (**d**) Positive staining of AGER protein in C33A cells (SP staining, ×400). (**e**) Negative control in Caski cells (SP staining, ×400). (**f**) Positive staining of AGER protein in Caski cells (SP staining, ×400). (**g**) Negative control in SiHa cells (SP staining, ×400). (**h**) Positive staining of AGER protein in SiHa cells (SP staining, ×400).

Initially, the localization of AGER protein in four cervical squamous cancer cells (SiHa, C33A, Caski, and MS751) was determined using immunocytochemical assay. As shown in Figure 1, AGER protein localized in the nucleus, cytoplasm and/or cytomembrane of the indicated cell lines, which was consistent with our previous report in cervical tissues [20]. SiHa, MS751 and C33A showed moderate or intense positive staining of AGER in cytomembrane, while there was no positive staining of AGER detected in cytomembrane of Caski cells.

### The mRNA and protein expression of AGER in cervical squamous cancer cell lines

**Figure 2:**
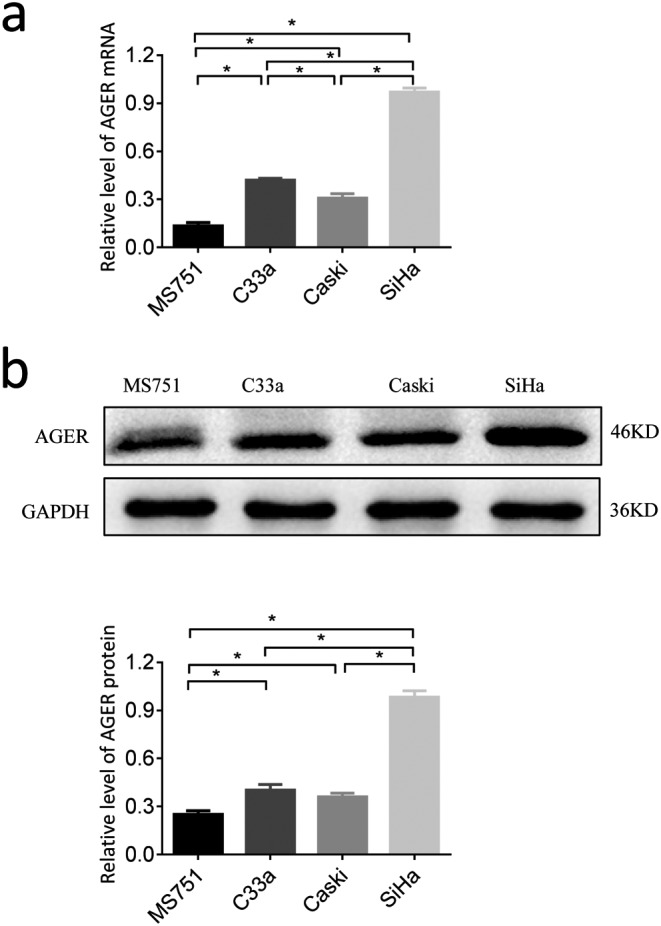
The expression of *AGER* mRNA and protein in human cervical squamous cancer cells (**a**) The mRNA levels of AGER in four cervical squamous cancer cells were detected by qRT-PCR. GAPDH transcript was used for normalization. (**b**) The protein levels of AGER in cervical squamous cancer cells was detected by Western blot. GAPDH protein level was used to validate equal sample loading. Data presented were mean ± S.D. from triplicate experiments (*P<0.05).

To determine whether AGER was overexpressed in cervical squamous cancer cell lines, the mRNA and protein levels of AGER were determined in MS751, C33A, SiHa, and Caski cells by qRT-PCR and Western blot, respectively. All these four cervical squamous cancer cell lines expressed certain mRNA levels as well as protein levels in AGER (Figure 2). The mRNA level of AGER was lowest in MS751 cells and highest in SiHa cells (*P*<0.05). Consistent with their mRNA levels, the expression of AGER protein was lowest in MS751 cells and highest in SiHa cells (*P*<0.05) (Figure 2).

### Effect of AGER on proliferation of cervical squamous cancer cells

**Figure 3:**
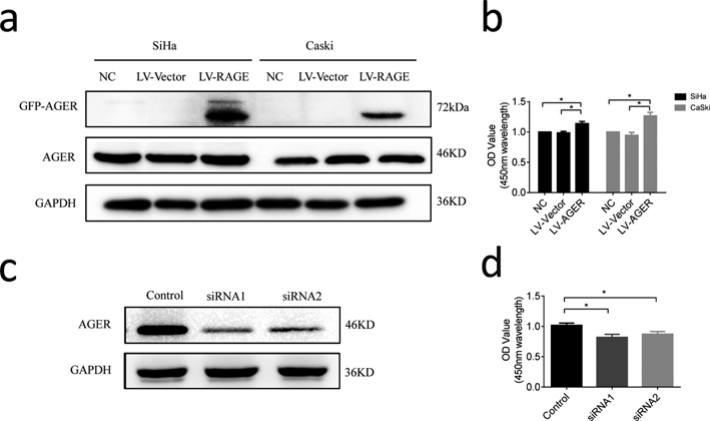
The effect of AGER on the proliferation of cervical cancer cells evaluated by CCK-8 assay (**a**) AGER cDNA and match vector were transfected into SiHa and Caski cells via lentivirus infection. Protein levels of AGER in AGER cDNA transfected, control vector transfected and NC cells by Western blot. GAPDH protein level was used to validate equal sample loading. (**b**) Cell proliferation was analyzed by CCK-8 assay. (**c**) Confirmation of AGER silencing in SiHa cells by Western blot. GAPDH protein level was used to validate equal sample loading. (**d**) Cell proliferation was analyzed by CCK-8 assay.

To understand whether AGER could affect biologic behavior in cervical squamous cancer cells, SiHa and Caski cell lines were first stably transfected with AGER cDNA via lentiviral infection. Ectopic expression of AGER was confirmed by Western blot assay. When compared with LV-vector cells (transfected with control vector) as well as negative control (NC) cells, LV-AGER cells (transfected with AGER cDNA) expressed a higher level of AGER (Figure 3a). Proliferation was then determined by CCK-8, as shown in Figure 3b (repeated three times), overexpression of AGER significantly enhanced the proliferation of SiHa and Caski cells compared with the control group (Figure 3b).

To confirm these results, we further analyzed the role of AGER by blocking its expression. AGER was silenced by two siRNAs (AGER-siRNA-1 and AGER-siRNA-2) in SiHa cell lines, in which the mRNA and protein level of AGER was the highest. Expectedly, as shown in Figure 3, transfection of cells with AGER siRNAs significantly suppressed AGER expression, which was confirmed by Western blot (Figure 3c). Silencing AGER significantly inhibited the cell proliferation in SiHa cells determined by CCK-8 assay (Figure 3d).

### Effect of AGER on apoptosis of cervical squamous cancer cells

**Figure 4:**
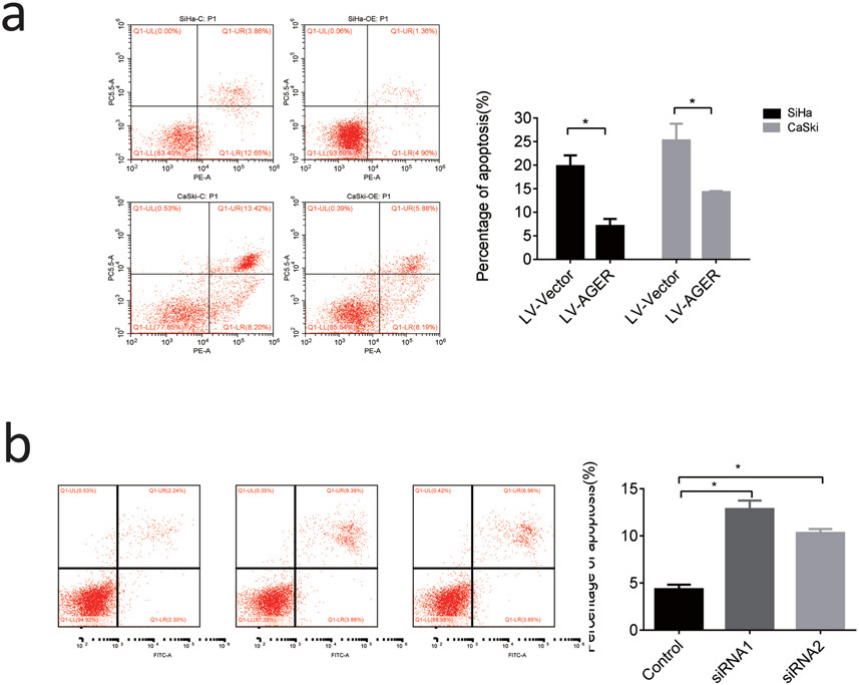
The effect of AGER on the apoptosis of cervical cancer cells (**a**) AGER cDNA and match vector were transfected into SiHa and Caski cells via lentivirus infection. Apoptosis percentage was analyzed by Annexin V-APC/7-AAD staining. (**b**) SiHa cells were transfected with AGER siRNA. Apoptosis percentage was analyzed by Annexin V-FITC/PI staining. Each bar represents mean ± S.D. of triplicate experiments.

Effect of AGER on apoptosis of cervical squamous cancer cells was further determined by flow cytometry assay. Up-regulating the expression of AGER significantly reduced the apoptosis percentage in SiHa cells as well as Caski cells (Figure 4a). Conversely, apoptosis percentage in SiHa/AGER-siRNA-1 cells and SiHa/AGER-siRNA-2 cells was significantly increased compared with SiHa/siRNA-NC cells (Figure 4b).

### Effect of AGER on migration of cervical squamous cancer cells

**Figure 5:**
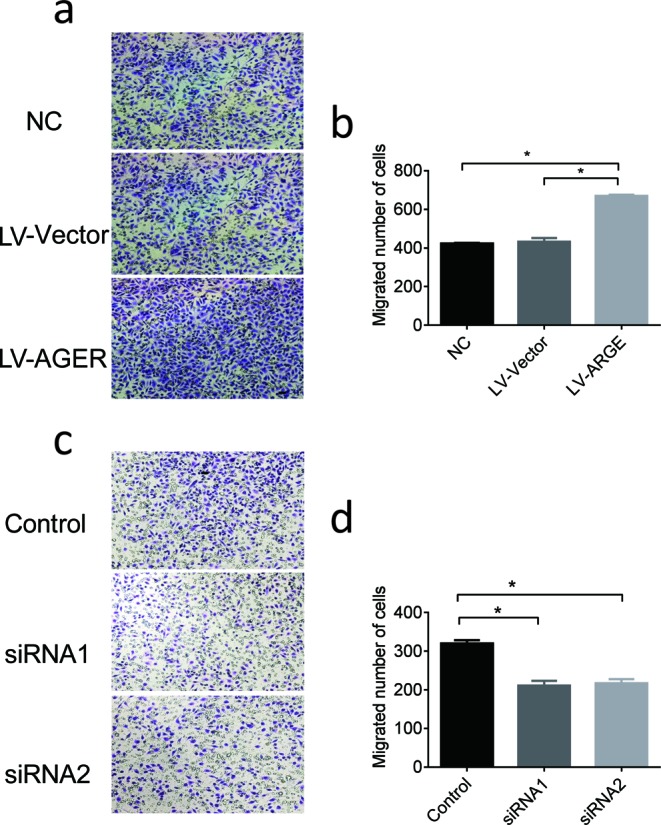
The effect of AGER on cervical cancer cells migration (**a**,**b**) AGER cDNA and match vector were transfected into SiHa cells via lentivirus infection. Cell migration was evaluated by transwell migration assay (×200 magnification). (**c**,**d**) SiHa cells were transfected with AGER siRNA. Cell migration was evaluated by transwell migration assay (×200 magnification). Data presented were mean ± S.D. from triplicate experiments (*P<0.05).

To explore the effect of AGER on the migration in cervical squamous cancer cells, transwell migration assay was performed. As shown in Figure 5, overexpression of AGER could significantly increase the number of SiHa cells that penetrated the membrane relative to control vector group (Figure 5a,b). Additionally, knockdown of AGER markedly reduced the number of SiHa cells that penetrated the membrane as compared with the control siRNA group (Figure 5c,d).

## Discussion

AGER is a multifunctional receptor that binds a broad repertoire of ligands, including AGEs, β-sheet fibrils, S100 protein family (S100B, S100P, S100A4, S100A6, S100A8/9, S100A11–13), high mobility group box-1 (HMGB1), and prions [10]. It plays decisive roles in diverse processes including inflammation and cancer [9,10]. AGER has been widely reported to be overexpressed in ovarian cancer [12], breast cancer [13], gastric cancer [14], colorectal cancer [21], and endometrial cancer [15]. With regard to cervical cancer, Xu et al. [22] reported that AGER 82G>S polymorphisms were associated with significantly elevated risk of cervical cancer. Along similar lines, our previous study documented that AGER protein expression was gradually increased from chronic cervicitis to cervical intraepithelial neoplasia and to squamous cervical cancer, and higher levels of AGER were related to histological differentiation [20]. In this investigation, we reported, for the first time, that AGER was positively expressed in a panel of squamous cervical cancer cells. Collectively, these data suggested that AGER may be implicated in the development and progression of cervical squamous cancer. To further elucidate its role in the progression of cervical squamous cancer, we silenced the expression of AGER by RNAi approach and overexpressed AGER via lentivirus infection. Our results showed that up-regulation of AGER in SiHa and Caski cells significantly promoted cancer cell growth, conversely, down-regulation of AGER expression in SiHa cells inhibited cell proliferation. These data are consistent with previous studies, which suggest AGER serve as an oncogenic gene in a wide spectrum of cancers [20]. AGER-deficient mice presented a decreased tendency for breast tumor growth [8] and AGER gene deletion inhibited the development of pancreatic intraepithelial neoplasia and progression to pancreatic ductal adenocarcinoma in a murine model [23]. Similar results were reported in endometrial cancer [15], colorectal cancer [18], and prostate cancer [24].

Mechanistically, Kwak et al. [25] reported that AGER knockout mice displayed striking impairment in breast tumor cell growth along with decreased mitogen-activated protein kinase signaling, tumor angiogenesis, and inflammatory cell recruitment. Elangovan et al. [24] documented that silencing AGER expression inhibited prostate tumor growth by activation of caspase-8 and caspase-3 death signaling and Radia et al. [16] reported that the blockage of AGER inhibited the proliferation of various subtypes of breast cancer via arresting cells in the G_1_ phase and inhibiting DNA synthesis. Our presented data indicated that AGER inhibits apoptosis in cervical squamous cancer. Apoptosis is well known as a protective mechanism against cancer progression by removing mutated, infected, or damaged cells. The development and progression of cancer are related to abnormal proliferation and apoptosis [26]. Thus, AGER may promote cervical squamous cancer growth via suppressing apoptosis.

It has also been well documented that AGER regulates cancer cell motility and drives tumor metastasis through its ligand [25]. Interaction of S100A4-AGER mediates S100A4-induced cell motility in colorectal cancer [17] and thyroid cancer [27]. AGER binding to S100A8/A9 promoted lung metastasis through actin polymerization and epithelial–mesenchymal transition in breast cancer [28]. AGER bound to S100A7 mediated its ability to activate extracellular signal regulated kinase, NF-κB, and cell migration [8]. Suppressing AGER reduced cell migration via the regulation of extracellular signal regulated kinase, and downstream pathways in human oral cancer cells [29]. *In vivo*, AGER neutralizing antibody significantly inhibited metastasis development in an established mouse model of lung metastasis [8] and silencing AGER using shRNA markedly reduced metastasis to the lung and liver in multiple xenograft and syngeneic breast cancer mouse models [25]. Nevertheless, the impact of AGER on migration in cervical cancer has not been elucidated so far. In the present study, overexpression of AGER in SiHa cells increased transwell migration. AGER knockdown with multiple siRNAs in SiHa cells led to decreased transwell migration. Thus, AGER may be a newly recognized factor regulating cancer cell migration and metastasis in cervical cancer. The underlying mechanism responsible for the effects of AGER on the motility of cervical cancer cells remains to be further clarified.

HPVs are considered the main etiological agents of cervical cancer, especially high-risk genotypes. Previously, Xu et al. [22] reported that AGER 82GS and 82SS genotype carriers in the HPV infection subgroup, but not in the HPV negative subgroup, had increased risk of cervical cancer compared with 82GG genotype. Their study indicated that AGER 82G>S polymorphisms, interacting with HPV infection, played an important role in the carcinogenesis of cervical cancer [22]. Additionally, previous bioinformatics analysis has indicated AGER contributed to prostate cancer cell proliferation by promoting Rb phosphorylation and degradation [19]. These results suggested AGER may be involved in HPV-induced cervical cancer. However, the involvement of synergistic effects of AGER and high-risk type HPV on the cervical carcinogenesis or progression need further study. Our future study will focus on the relationship between HPV and AGER in cervical cancer as well as the mechanism of AGER involved in HPV-caused cervical cancer.

## Conclusion

Overexpression of AGER promotes and silencing its expression suppresses the proliferation and migration of cervical cancer cells. Our current data provide novel evidence for a potential role of AGER in bridging HPV-induced inflammation and cervical cancer. However, the molecular mechanism responsible for the role of AGER in such malignancy and the involvement of synergistic effects of AGER and HPV on the squamous cervical carcinogenesis or progression needs further investigation.

## Abbreviations

AGE: advanced glycation end product
AGER: AGE-specific receptor
HPV: human papillomavirus
NC: negative control
qRT-PCR: quantitative real-time PCR
TBST: TBS with tween
