# Expression of Concern: AGER promotes proliferation and migration in cervical cancer

**DOI:** 10.1042/BSR20171329_EOC

**Published:** 2026-08-14

**Authors:** Xuejie Zhu, Lulu Zhou, Ruyi Li, Qi Shen, Huihui Cheng, Zongji Shen, Haiyan Zhu

**Affiliations:** 1 Department of Obstetrics and Gynecology, The First Affiliated Hospital of Soochow University, Suzhou, China; 2 Department of Obstetrics and Gynecology, The Second Affiliated Hospital of Wenzhou Medical University, Wenzhou, China

**Keywords:** AGER, cervical cancer, migration, proliferation

*Bioscience Reports* has been made aware of potential issues surrounding the scientific validity of this paper and is hence issuing an Expression of Concern to notify readers.

A duplication between the Figure 5a NC and LV-Vector panels was identified by the authors during internal self-inspection in October 2023 and an Erratum application was submitted to the Editorial Office.

The Editorial Office notes that the western blots have white backgrounds with very high contrast and exposure. The authors have provided raw data related to Figure 5 and cropped western blot images for verification, but the complete uncropped original western blot membrane scans are not available. The authors state that this figure misplacement error does not affect the study's core experimental data, results or overall conclusions.

A corrected version of Figure 5 is available from the authors upon request.

As concerns remain surrounding the western blots, the Editorial Board has decided that the article will remain published but with an associated Expression of Concern.

